# Sex‐Specific Biological Predictors for Identifying Individuals With Low Bone Mineral Density Using the Taiwan Biobank Database

**DOI:** 10.1155/joos/6485124

**Published:** 2026-06-23

**Authors:** Yu-Hsu Chen, Yu-Pao Hsu, Ming-Te Cheng, Shau-Kwaun Chen, Jia-Wen Guo

**Affiliations:** ^1^ Department of Orthopedic Surgery, Ministry of Health and Welfare, Taoyuan General Hospital, Taoyuan, Taiwan, tygh.gov.tw; ^2^ Department of Biology and Anatomy, National Defense Medical University, Taipei, Taiwan; ^3^ Department of Orthopedics, Tri-Service General Hospital, National Defense Medical University, Taipei, Taiwan, tsgh.ndmctsgh.edu.tw; ^4^ Department of Biomedical Engineering, Chung Yuan Christian University, Taoyuan, Taiwan, cycu.edu.tw; ^5^ Department of Medical Science, National Tsing Hua University, Hsinchu, Taiwan, nthu.edu.tw; ^6^ Institute of Neuroscience, National Chengchi University, Taipei, Taiwan, nccu.edu.tw; ^7^ College of Nursing, University of Utah, Salt Lake City, Utah, USA, utah.edu

## Abstract

Osteoporosis has seen a rapid rise in Taiwan due to its aging population. The pathogenesis of low bone mineral density (BMD) differs between women and men, highlighting the critical need to explore sex‐specific biological predictor variables—an area with significant knowledge gaps. This study aimed to identify sex‐specific predictors using a population database through machine learning. We conducted a retrospective cross‐sectional study of 19,868 individuals aged 40 and above from the Taiwan Biobank database who had no cancer diagnosis and underwent BMD examinations between 2020 and 2022, with available demographic, laboratory, and physical examination data. The Extreme Gradient Boosting (XGBoost) model was employed to identify patterns of predictor variables for low BMD. SHapley Additive exPlanations (SHAP) were applied to visualize and interpret the contribution of each predictor to low BMD in women and men. Among participants, 63% (*n* = 12,565) were women and 44% had low BMD (35% osteopenia and 9% osteoporosis), with a mean age of 54 years (SD = 9.6; range 40–88). The area under the curve values were 0.75 for women and 0.63 for men. Age, postmenopausal status, and BMI were the most significant predictors of low BMD in women, whereas age, BMI, and waist circumference were the strongest predictors in men. In addition, hematological factors (WBC, RBC, platelet count, hemoglobin), metabolic indicators (body fat percentage, triglycerides, and blood pressure), uric acid, and liver (gamma‐GT) and renal (eGFR) function markers were also associated with BMD. These predictors differed in their relative importance between women and men, highlighting clear sex‐specific patterns in factors associated with low BMD. Recognizing these determinants is essential for prevention and early intervention strategies, while further research is needed to better understand the complex mechanisms underlying bone metabolism.

## 1. Introduction

Osteoporosis is a systemic skeletal disorder characterized by reduced bone mass and deterioration of bone tissue, leading to an increased risk of fractures and posing a major public health concern in aging populations. Its prevalence has risen rapidly in Taiwan and across Asia due to population aging. According to the Asia‐Pacific Regional Audit by the International Osteoporosis Foundation and epidemiological data from Taiwan, individuals aged ≥ 50 years accounted for 32% of the population in 2013, with projections increasing to 42% by 2025 and 57% by 2050 [1].

Given its substantial impact on health and quality of life, identifying factors associated with low bone mineral density (BMD) is essential for early risk detection. Aging is strongly associated with reduced bone mass and strength due to decreased osteoblast activity and increased osteoclast activity [2]. Body composition also plays a complex role; while higher body weight may increase BMD through mechanical loading, visceral adiposity has been linked to lower BMD [2, 3]. Importantly, these relationships differ by sex. Hormonal changes, particularly estrogen deficiency during menopause, accelerate bone resorption and increase fracture risk in women, whereas bone loss in men tends to occur more gradually and is influenced by different metabolic and hormonal pathways [4].

Beyond these established factors, emerging evidence suggests that a wide range of biological variables are associated with BMD. Hematological parameters, including hemoglobin, white blood cells (WBC), red blood cells (RBCs), and platelet counts, may reflect interactions between hematopoiesis and bone metabolism [5–10]. In addition, metabolic factors such as body fat percentage, triglycerides, blood pressure, and uric acid, as well as liver and renal function markers (e.g., gamma‐glutamyl transferase [gamma‐GT] and estimated glomerular filtration rate [eGFR]), may influence bone health through complex physiological interactions [2, 4, 11, 12].

Despite these findings, important knowledge gaps remain regarding how these factors differ between women and men. The pathogenesis of low BMD is influenced by sex‐specific biological mechanisms, yet most studies have not comprehensively examined these differences across multiple physiological systems. Furthermore, traditional statistical approaches may not fully capture the complex relationships among these predictors. Using large‐scale population‐based data provides an opportunity to capture real‐world variability, improve generalizability, and identify subtle patterns across diverse biological factors. Therefore, this study aimed to identify sex‐specific biological predictors of low BMD using machine learning approaches in a large Taiwanese population, with the goal of supporting more targeted prevention and earlier intervention.

## 2. Materials and Methods

### 2.1. Study Design

A retrospective, cross‐sectional study was conducted utilizing a cohort derived from the Taiwan Biobank, a comprehensive biological research database [13].

### 2.2. Dataset and Cohort Selection

Taiwan Biobank is a government‐supported research database that has collected phenotypic and genomic data from the Taiwanese population since 2012. The database includes demographic, laboratory, and physical examination data. Participation is voluntary, and all participants provide written informed consent at recruitment for the collection and use of their data and biospecimens for research purposes. Data collection and management are conducted in accordance with relevant ethical approvals and data governance regulations [13].

The cohort for this study consisted of Taiwanese individuals aged ≥ 40 years without a self‐reported history of cancer who participated in the Taiwan Biobank between 2020 and 2022. Biological variables obtained from blood tests provided objective indicators of physiological processes and may offer additional insights into bone health, supporting their use in identifying clinically relevant predictors of low BMD.

We initially considered including lifestyle and clinical factors known to affect bone health, such as medication use, smoking, physical activity, and alcohol consumption, to provide a more comprehensive assessment. However, detailed medication data were not available in the Taiwan Biobank, and the available lifestyle variables (e.g., smoking, exercise, and alcohol use) had limited data quality due to incomplete responses, as data collection relied on voluntary self‐reported questionnaires. Additionally, some survey items were ambiguously defined. For example, in the question assessing current exercise habits, the response options were: *1 = Yes, engages in regular exercise; 2 = No, does not engage in regular exercise*. However, these options lacked clear definitions, allowing for individual variation in the interpretation of the term “regular.” As a result, these variables were excluded from the analysis based on data availability and quality concerns.

To maintain analytical rigor, we focused on variables with standardized collection procedures and high completeness across participants. The cohort selected for this study consisted of participants who provided complete demographic, laboratory, and physical examination data, including variables such as age, sex, blood pressure, body fat percentage, waist circumference, glycated hemoglobin (HbA1c), hemoglobin, triglycerides, high‐density lipoprotein cholesterol (HDLc), low‐density lipoprotein cholesterol (LDLc), total bilirubin, uric acid, eGFR, gamma‐GT, serum glutamic oxaloacetic transaminase/serum glutamic pyruvic transaminase (SGOT/SGPT) ratio, platelet count, RBC, WBC, postmenopausal status (for women participants), and BMD T‐score. All variables of interest in this study were collected on the same day for each participant during their visit to the Taiwan Biobank data collection site. This temporal consistency enhances the validity of the identified biological predictor variables for low BMD.

In the Taiwan Biobank, BMD measurements are obtained at the heel (calcaneus) of the nondominant foot using an Achilles InSight ultrasound system (GE, Madison, WI), with results expressed in g/cm^2^ and used to derive T‐scores; this ultrasound‐based approach is predefined by the Taiwan Biobank protocol and does not involve dual‐energy X‐ray absorptiometry (DXA). Using DXA‐derived T‐scores at the hip or spine, which are considered the gold standard diagnostic sites, osteoporosis is conventionally defined as a T‐score ≤ −2.5, while osteopenia is defined as a T‐score between −1.0 and −2.5 at these same sites [14, 15]. Calcaneal ultrasound assesses peripheral bone, and its T‐scores are not directly comparable to those obtained by DXA. Therefore, these measurements are more appropriately interpreted as screening indicators rather than definitive diagnostic criteria.

The initial pool of this study consisted of 21,898 individuals. After excluding those with missing data (*n* = 1213) and outliers (*n* = 817) to minimize the influence of extreme values, a total of 19,868 individuals were included in the final analysis. Outliers were defined as observations with absolute z‐scores > 3, values beyond 1.5 × IQR, or extreme values based on Mahalanobis distance, as implemented in the check_outliers() function from the *performance* R package [16, 17].

### 2.3. Data Analysis

Participants were classified into low and normal BMD groups. Descriptive analyses, univariate logistic regression, and independent‐samples *t*‐tests were performed to describe and assess group differences. All the variables of interest were included in machine learning models (support vector machine, decision tree, random forest, and Extreme Gradient Boosting [XGBoost]) to identify predictors of low BMD. The analyses were conducted using R (version 4.5.2), with the packages including *xgboost, SHAPforxgboost, e1071, kernlab, randomForest,* and *rpart.* The dataset was randomly divided into training (70%) and test (30%) sets. Model development and hyperparameter tuning were performed using grid search with k‐fold cross‐validation within the training set to improve robustness and prevent potential overfitting. Final model performance was evaluated on the independent test set.

Model performance was assessed using the area under the receiver operating characteristic curve (AUC), precision–recall AUC, sensitivity, and specificity. Sensitivity analyses comparing model performance with and without outlier exclusion showed negligible differences (ΔAUC < 0.01), with unchanged model rankings, indicating robust results. The optimal model was selected based on overall performance. Model calibration was assessed using the Brier score (range: 0‐1) and the Hosmer–Lemeshow test. Lower Brier scores and nonsignificant Hosmer–Lemeshow test results indicate better calibration. Decision curve analysis was conducted to determine the clinical applicability of the models.

SHapley Additive exPlanations (SHAP) summary plots were employed to elucidate the ranking of variables/predictors and their influence on the model output. We generated SHAP contribution dependency plots for the most influential predictor variables to visualize their relationships, including any nonlinear patterns with low BMD [18]. The SHAP values above 0 indicate an increased likelihood of predicting the outcome variable as 1, which signifies low BMD, compared to 0, representing normal BMD. These positive SHAP values suggest a higher risk of having low BMD. SHAP dependence plots were utilized to identify approximate thresholds of individual predictor variables associated with an increased likelihood of low BMD. The R version and session information are provided in the Supporting Information.

### 2.4. Ethics Approval

Ethical approval for this study was obtained from the Research Ethics Committee of National Chengchi University (IRB No. NCCU‐REC‐201904‐I017). The study was conducted using the Taiwan Biobank resource under application number TWBR11308‐07. Participants in the Taiwan Biobank provided written informed consent at enrollment for the collection and use of their data and biospecimens for research purposes.

## 3. Results

Of the eligible sample (*N* = 19,868), 12,565 (63%) were women. In this sample, the mean age was 54 (SD = 9.6, ranging from 40 to 88 years), with 44% having low BMD (35% with osteopenia and 9% with osteoporosis). Descriptive statistics for each variable by BMD status in women and men are presented in Table 1. Among women, most selected variables differed significantly between normal and low BMD (*p* < 0.05), except for LDL‐C and the SGOT/SGPT ratio. Among men, age, RBC, platelet count, hemoglobin, BMI, body fat percentage, LDL‐C, SGOT/SGPT ratio, uric acid, and eGFR differed significantly between normal and low BMD (*p* < 0.05).

**TABLE 1:** Descriptive characteristics of women and men by BMD status.

**Women (*N* = 12,565)**						
		**Normal *n* = 7326**	**Low BMD *n* = 5239**			
**Variable**	**Group**	***n* (%)**	***n* (%)**	**OR**	**95% OR**	**p**

Menopause	Yes	3158 (43.1)	4219 (80.5)	5.46	5.03, 5.93	< 0.001
	No	4168 (56.9)	1020 (19.5)			

	*Unit*	*M (SD)*	*M (SD)*	*t*	*df*	*p*
Age	Year	50.99 (8.24)	58.63 (8.70)	−50.06	12,563	< 0.001
RBC	10^6^/μL	4.57 (0.44)	4.55 (0.46)	3.29	12,563	0.001
WBC	10^3^/μL	5.52 (1.58)	5.33 (1.46)	6.76	12,563	< 0.001
Platelet	10^3^/μL	253.45 (64.72)	235.89 (60.18)	15.44	12,563	< 0.001
Hemoglobin	g/dL	13.10 (1.33)	13.23 (1.17)	−5.58	12,563	< 0.001
BMI	Kg/m^2^	24.10 (3.79)	23.35 (3.53)	11.2	12,563	< 0.001
Body fat rate	%	33.14 (6.23)	32.38 (6.06)	6.89	12,563	< 0.001
Waist circumference	cm	81.68 (9.49)	81.27 (9.21)	2.4	12,563	0.016
HbA1c	%	5.70 (0.50)	5.81 (0.55)	−11.51	12,563	< 0.001
SBP	mmHg	119.45 (18.37)	125.12 (20.07)	−16.4	12,563	< 0.001
DBP	mmHg	73.31 (10.96)	74.15 (10.88)	−4.24	12,563	< 0.001
Triglyceride	mg/dL	104.86 (62.61)	109.66 (63.48)	−4.21	12,563	< 0.001
HDLc	mg/dL	60.47 (13.48)	61.21 (13.79)	−3.01	12,563	0.003
LDLc	mg/dL	123.03 (32.54)	123.59 (32.94)	−0.95	12,563	0.341
Total bilirubin	mg/dL	0.61 (0.24)	0.65 (0.23)	−7.44	12,563	< 0.001
Gamma‐GT	unit/L	19.24 (16.22)	20.23 (16.64)	−3.32	12,563	0.001
SGOT/SGPT ratio		1.18 (0.45)	1.17 (0.42)	1.14	12,563	0.254
Uric acid	mg/dL	4.83 (1.09)	4.88 (1.12)	−2.5	12,563	0.013
eGFR	mL/min/1.73 m^2^	107.65 (10.36)	102.49 (11.26)	26.57	12,563	< 0.001

**Men (*N* = 7303)**						
		**Normal *n* = 3786**	**Low BMD *n* = 3517**			
	**Unit**	**M (SD)**	**M (SD)**	***t***	***df***	***p***

Age	Year	52.98 (9.71)	56.82 (10.03)	−16.63	7301	< 0.001
RBC	10^6^/μL	5.12 (0.50)	5.04 (0.51)	6.68	7301	< 0.001
WBC	10^3^/μL	5.65 (1.54)	5.69 (1.54)	−1.30	7301	0.193
Platelet	10^3^/μL	227.10 (54.57)	221.73 (55.40)	4.17	7301	< 0.001
Hemoglobin	g/dL	15.14 (1.09)	14.96 (1.15)	7.00	7301	< 0.001
BMI	Kg/m^2^	25.87 (3.39)	25.34 (3.43)	6.64	7301	< 0.001
Body fat percentage	%	23.49 (4.90)	23.07 (5.05)	3.60	7301	< 0.001
Waist circumference	cm	89.15 (8.96)	88.97 (9.17)	0.85	7301	0.394
HbA1C	%	5.85 (0.62)	5.87 (0.62)	−1.25	7301	0.211
SBP	mmHg	130.52 (17.34)	131.10 (17.70)	−1.43	7301	0.153
DBP	mmHg	80.28 (10.84)	80.43 (10.96)	−0.61	7301	0.543
Triglyceride	mg/dL	134.49 (78.37)	135.17 (80.56)	−0.37	7301	0.714
HDLc	mg/dL	49.47 (11.27)	49.15 (11.26)	1.21	7301	0.227
LDLc	mg/dL	122.39 (32.23)	120.42 (33.40)	2.56	7301	0.01
Total bilirubin	mg/dL	0.77 (0.29)	0.76 (0.29)	1.94	7301	0.053
Gamma‐GT	unit/L	28.05 (20.93)	27.90 (20.83)	0.31	7301	0.755
SGOT/SGPT ratio		0.94 (0.35)	0.97 (0.39)	−3.98	7301	< 0.001
Uric acid	mg/dL	6.32 (1.27)	6.19 (1.33)	4.26	7301	< 0.001
eGFR	mL/min/1.73 m^2^	98.78 (13.52)	96.85 (13.93)	6.02	7301	< 0.001

*Note:* The reference range for each variable was determined according to the normal values provided by the Taiwan Biobank [13].

Abbreviations: BMD, bone mineral density; DBP, diastolic blood pressure; eGFR, estimated glomerular filtration rate; Gamma‐GT, gamma‐glutamyl transpeptidase; HbA1c, glycated hemoglobin; HDLc, high‐density lipoprotein cholesterol; LDLc, low‐density lipoprotein cholesterol; OR, odds ratio; RBC, red blood cell count; SBP, systolic blood pressure; SGOT/SGPT ratio, serum glutamic oxaloacetic transaminase/serum glutamic pyruvic transaminase ratio; WBC, white blood cell count.

The XGBoost models demonstrated superior predictive performance, achieving an AUC of 0.75 (95% CI: 0.74–0.77), accuracy of 0.70, sensitivity of 0.67, and specificity of 0.73 in women, compared with an AUC of 0.62 (95% CI: 0.61–0.66), accuracy of 0.60, sensitivity of 0.53, and specificity of 0.65 in men. Calibration was acceptable, with Brier scores of 0.20 in women and 0.24 in men, and nonsignificant Hosmer–Lemeshow test results (women: *p* = 0.37; men: *p* = 0.07), indicating good agreement between predicted and observed outcomes.

SHAP analysis revealed both shared and sex‐specific patterns of feature importance between women and men. In women, age, menopausal status, and metabolic factors (including BMI and body fat percentage) were the most important predictors of low BMD, with higher SHAP values compared to other variables. Hematological parameters, including hemoglobin, WBC count, and platelet count, also contributed to model predictions. In addition, eGFR and gamma‐GT were identified as relevant predictors (Figure 1a). In men, age was also the most important predictor of low BMD. Metabolic factors, including lower BMI and body fat percentage, as well as higher waist circumference and triglyceride levels, showed relatively strong contributions to the model. Uric acid, a known antioxidant, was an important predictor, with lower levels associated with a higher predicted risk of low BMD. eGFR also contributed to the model. Moreover, hematological variables, including hemoglobin and RBC count, along with liver‐related variables such as gamma‐GT and the SGOT/SGPT ratio, were also identified as relevant predictors (Figure 2a).

FIGURE 1SHAP explanations for visualizing XGBoost model for low BMD in women. (a) In the SHAP summary plot, color indicates the magnitude of feature values, with purple representing higher values and yellow representing lower values. The *x*‐axis represents the SHAP value, reflecting the impact on the model output (log‐odds of low BMD). Positive SHAP values indicate an increase in the predicted log‐odds of low BMD, whereas negative values indicate a decrease. For example, postmenopausal status is coded as 1 (present) and 0 (absent), corresponding to purple and yellow, respectively. Purple points located to the right of zero (SHAP > 0) indicate that the presence of postmenopausal status is associated with higher predicted log‐odds of low BMD, whereas yellow points to the left of zero (SHAP < 0) indicate that the absence of postmenopausal status is associated with lower predicted log‐odds. (b) SHAP dependence plots are shown for the top 12 predictors in the model. The *x*‐axis represents the value of each predictor, and the *y*‐axis shows the SHAP value, reflecting the impact on the model output (log‐odds of low BMD). The red solid line illustrates the overall relationship between each predictor and its SHAP value across the sample, while each gray point represents an individual observation. The red dashed horizontal line marks SHAP = 0, indicating no effect on the predicted outcome. Values above this line indicate an increase in the predicted log‐odds of low BMD, whereas values below indicate a decrease. For example, in the age plot, the red solid line crosses zero at approximately the mid‐50s and shows a positive linear trend, indicating that increasing age is associated with a higher predicted risk of low BMD.

**Figure:**
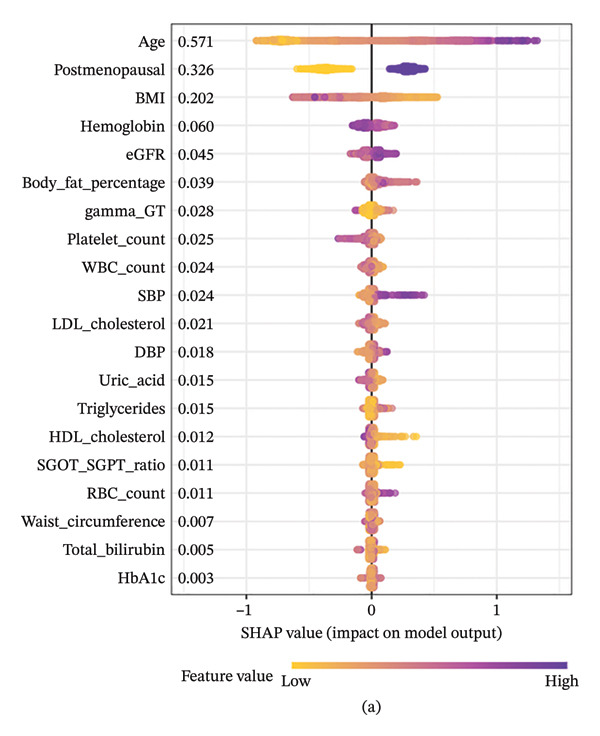

**Figure:**
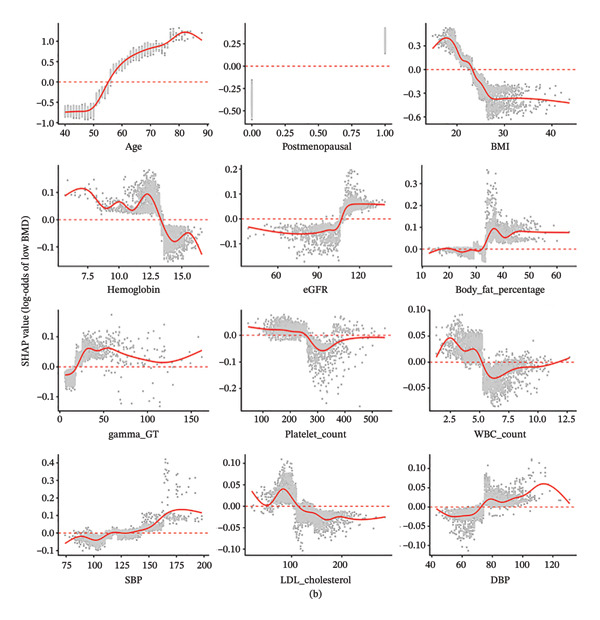

FIGURE 2SHAP explanations for visualizing XGBoost model for low BMD in men. (a) In the SHAP summary plot, color indicates the magnitude of feature values, with purple representing higher values and yellow representing lower values. The *x*‐axis represents the SHAP value, reflecting the impact on the model output (log‐odds of low BMD). Positive SHAP values indicate an increase in the predicted log‐odds of low BMD, whereas negative values indicate a decrease. For example, higher age is represented in purple and lower age in yellow. Purple points located to the right of zero (SHAP > 0) indicate that higher age is associated with higher predicted log‐odds of low BMD, whereas yellow points to the left of zero (SHAP < 0) indicate that lower age is associated with lower predicted log‐odds. (b) SHAP dependence plots are shown for the top 12 predictors in the model. The *x*‐axis represents the value of each predictor, and the *y*‐axis shows the SHAP value, reflecting the impact on the model output (log‐odds of low BMD). The red solid line illustrates the overall relationship between each predictor and its SHAP value across the sample, while each gray point represents an individual observation. The red dashed horizontal line marks SHAP = 0, indicating no effect on the predicted outcome. Values above this line indicate an increase in the predicted log‐odds of low BMD, whereas values below indicate a decrease. For example, in the age plot, the red solid line crosses zero at approximately 50 years and shows a positive linear trend, indicating that increasing age is associated with a higher predicted risk of low BMD.

**Figure:**
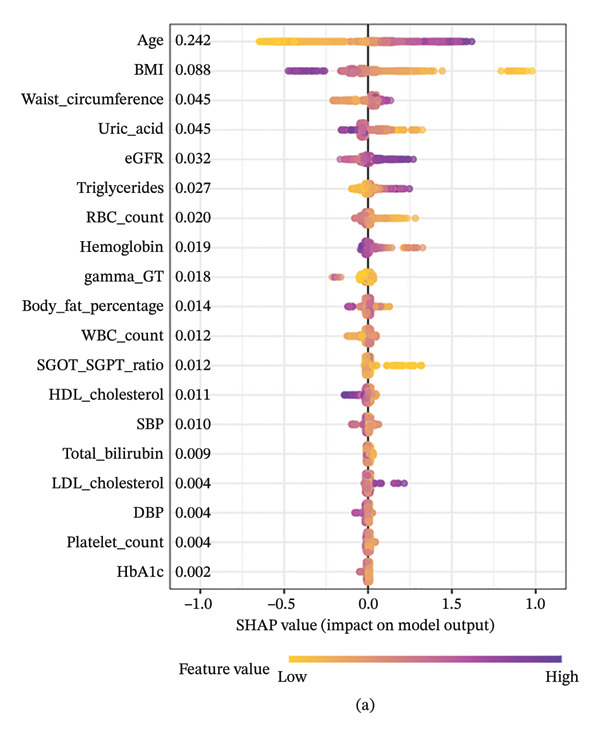

**Figure:**
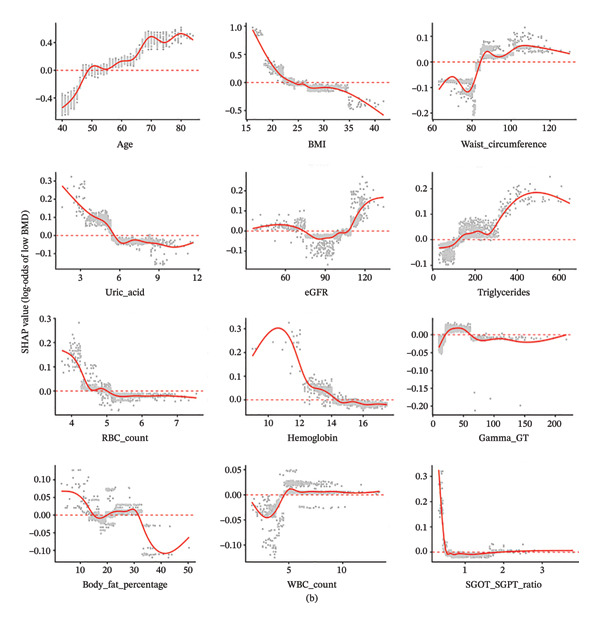

Several predictors were shared between women and men, including age, BMI, body fat percentage, hematological variables, gamma‐GT, and eGFR. However, distinct sex‐specific patterns were observed. In women, menopausal status emerged as a unique and dominant predictor, highlighting the role of hormonal factors. In contrast, men showed stronger contributions from a more multifactorial profile, including metabolic variables (i.e., waist circumference and triglycerides), uric acid, and hematological variables. To examine how model predictions vary with the values of important predictors across the entire dataset, we used SHAP dependence plots focusing on the top 12 variables in both the women’s (Figure 1b) and men’s models (Figure 2b). Based on SHAP dependence analysis of the top 12 predictors in the women’s model, features were grouped into two patterns according to their association with low BMD. The positive linear‐like pattern included variables for which higher values were associated with higher predicted log‐odds of low BMD. Age showed an increasing pattern beginning in the mid‐50s, while body fat percentage increased notably above ∼33%. Blood pressure also showed a similar pattern, with SBP above ∼150 mmHg and DBP above ∼75 mmHg associated with higher predicted log‐odds. Higher values of eGFR (> ∼105 mL/min/1.73 m^2^) and gamma‐GT (> ∼15 unit/L) were also associated with higher predicted log‐odds. Postmenopausal status was similarly associated with higher predicted log‐odds of low BMD. In contrast, the inverse linear‐like pattern included variables for which lower values were associated with higher predicted log‐odds of low BMD. These included BMI (< 24), hemoglobin (< ∼13.5 g/dL), platelet count (< ∼250 × 10^3^/μL), WBC count (< ∼5.4 × 10^3^/μL), and LDLc (< ∼110 mg/dL).

For the men’s model, the top 12 predictors were grouped into three patterns. The positive linear‐like pattern included age (> ∼47 years), waist circumference (> ∼85 cm), triglycerides (> ∼120 mg/dL), and WBC count (> ∼4.2 × 10^3^/μL), where higher values were associated with higher predicted log‐odds of low BMD. The inverse linear‐like pattern included BMI (< ∼24), uric acid (< ∼5.5 mg/dL), RBC count (< ∼4.5 × 10^6^/μL), hemoglobin (< ∼14.2 g/dL), body fat percentage (< ∼33%), and SGOT/SGPT ratio (< ∼0.5), where lower values were associated with higher predicted log‐odds. A nonlinear pattern was observed for eGFR and gamma‐GT. eGFR showed a U‐shaped association, with higher predicted log‐odds at both low (< ∼75 mL/min/1.73 m^2^) and high (> ∼105 mL/min/1.73 m^2^) levels. Gamma‐GT showed a fluctuating pattern, with higher predicted log‐odds mainly observed between ∼20 and ∼60 unit/L.

**FIGURE 3:**
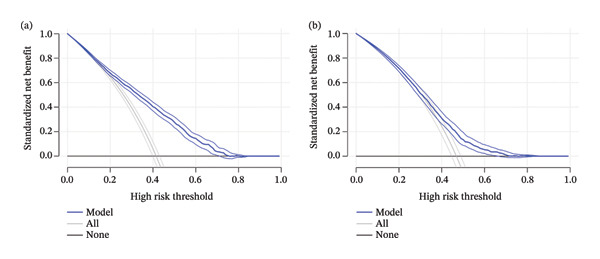
Decision curve analysis (DCA) curves of XGBoost models for low BMD in women (a) The blue line lies above both the gray (treat‐all) and black (treat‐none) lines, indicating that the model is clinically useful across that threshold range (approximately 0.1–0.7), where clinicians would consider screening for low BMD. The benefit was greatest at moderate thresholds (around 0.2–0.5). (b) The men’s model (blue line) showed greater net benefit than the treat‐all (gray) and treat‐none (black) strategies across clinically relevant thresholds (approximately 0.1–0.6), at which clinicians would consider screening for low BMD. The benefit was greatest at moderate thresholds (around 0.3–0.5).

Decision curve analysis showed that both the female and male models provided greater net benefit than the “treat‐all” and “treat‐none” strategies across clinically relevant thresholds (Figure 3). Compared with the women’s model (approximately 0.1–0.7), the men’s model showed benefit over a slightly narrower range (approximately 0.1–0.6). Overall, these findings indicate sex‐specific patterns of associated variables and suggest that the models are useful for guiding BMD screening decisions, particularly at moderate thresholds.

## 4. Discussion

In this study, we used machine learning to identify predictors of low BMD separately in women and men using data from the Taiwan Biobank. Age and BMI were the strongest predictors in both sexes, while postmenopausal status was particularly important in women. These results are consistent with established evidence linking aging, body composition, and menopause to BMD, and they also show that these factors play a central role in predicting low BMD in this population [2, 3, 19].

The patterns of predictors differed between women and men. In women, age, postmenopausal status, and BMI accounted for a substantial proportion of the predictive performance. In contrast, in men, age was the primary predictor, followed by a more dispersed pattern with contributions from metabolic, hematological, and other variables. This may partly explain the lower predictive performance in men, suggesting that key factors of bone health in men may not be adequately captured. Factors such as sex hormones, physical activity, and lifestyle behaviors likely play an important role [12] but were not included due to limitations in the Taiwan Biobank data [13]. Specifically, these variables have limited details and lack standardized definitions, restricting their ability to contribute meaningfully to the analysis. Their exclusion may have contributed to the reduced predictive performance observed in the men’s model.

Hematological variables, including WBC, RBC, platelet count, and hemoglobin, were consistently identified as relevant predictors, with clear differences between sexes. WBC was associated with low BMD in both sexes, but in opposite directions. Platelet count was relevant only in women, whereas RBC count was specific to men. Lower hemoglobin levels were associated with low BMD in both sexes, with different thresholds. These findings suggest a link between hematological measures and bone health. Similar associations have been reported in previous studies [7, 9, 10, 20–22]. Biological evidence indicates that platelets may support bone formation through the release of growth factors [21], while RBCs contribute through oxygen delivery and signaling processes related to osteogenesis [22]; correspondingly, anemia and iron deficiency have been associated with low BMD [10]. While existing studies have suggested potential mechanisms, further research is needed to clarify their roles and clinical relevance across sexes.

Metabolic variables, including blood pressure, triglycerides, body fat percentage, and waist circumference, were important predictors, with differing importance between women and men. These findings support the link between metabolic health and bone health [19], and suggest that these relationships may vary by sex. In addition, uric acid was identified as an important predictor in men. As an endogenous antioxidant, it may influence bone metabolism by reducing oxidative stress; lower levels may therefore be associated with lower BMD [23].

Liver‐related markers, including the SGOT/SGPT ratio and gamma‐GT, were also associated with low BMD in our models. A lower SGOT/SGPT ratio was linked to low BMD in men, which may reflect the liver’s role in metabolism, including calcium regulation [2] and growth hormone–mediated osteoblast activity [12]. Gamma‐GT was associated with lower BMD in both sexes, as reported in previous studies [24, 25], although the mechanisms linking GGT to bone health remain unclear.

eGFR was also associated with low BMD, with patterns differing by sex. In women, higher eGFR was associated with lower BMD, whereas in men, a U‐shaped relationship was observed. However, this association should be interpreted with caution, as it may be confounded by factors such as age, BMI, and menopausal status [11, 26]. Together, these findings highlight the complex roles of liver‐ and kidney‐related markers in bone metabolism.

Overall, this study shows that predictors of low BMD differ between women and men and involve multiple biological mechanisms. These findings should be interpreted as predictive rather than causal.

### 4.1. Limitations

Several limitations should be acknowledged. First, this study is based on retrospective, cross‐sectional data and is restricted to variables available in the database, which may introduce bias and limit generalizability and causal inference. Nevertheless, we incorporated a broad range of biologically relevant variables, including liver, renal, hematological, and metabolic domains, providing a comprehensive evaluation of potential predictors in both sexes. Second, the models showed modest predictive performance, particularly in men. Although performance was higher in women, sensitivity may still be suboptimal for screening. This may reflect heterogeneity in associated factors and the absence of key predictors, highlighting the need for further model refinement. Third, BMD was assessed using calcaneal ultrasound rather than DXA. Accordingly, the derived T‐scores are not directly comparable to DXA‐based measures and should be interpreted as screening estimates rather than definitive diagnoses. As this method evaluates peripheral skeletal sites, it may not accurately reflect BMD at central sites such as the hip and spine. Therefore, external validation in independent cohorts, particularly using DXA‐based BMD measurements, is warranted to confirm the generalizability and clinical utility of our findings. Fourth, the exclusion of participants with incomplete records and outliers may have introduced additional bias; however, the proportion excluded was small, and sensitivity analyses indicated minimal impact on the results. Finally, the absence of key factors influencing bone health, such as smoking status, physical activity, and medication use, remains a limitation. Future studies incorporating more detailed lifestyle and clinical data are needed to better elucidate the determinants of low BMD.

## 5. Conclusion

This study identified distinct, sex‐specific predictors of low BMD in a Taiwanese population using machine learning. These results highlight sex‐specific differences in biological factors associated with low BMD and underscore the need for tailored approaches to risk assessment. These findings support targeted prevention, earlier interventions, and further research in bone health.

## Funding

This study was funded by Tao Yuan General Hospital, Ministry of Health and Welfare, 10.13039/100015820, PTH110061 PTH110065.

## Conflicts of Interest

The authors declare no conflicts of interest.

## Supporting Information

Additional supporting information can be found online in the Supporting Information section.

## Supporting information


**Supporting Information** R version and session information used for the analyses.

## Data Availability

The data that support the findings of this study are not publicly available due to restrictions, as they were obtained under license from the Taiwan Biobank (https://www.twbiobank.org.tw/). However, the data are available from the authors upon reasonable request and with permission from the Taiwan Biobank.
